# Risk Factors for Maternal and Fetal Mortality in Acute Fatty Liver of Pregnancy and New Predictive Models

**DOI:** 10.3389/fmed.2021.719906

**Published:** 2021-08-05

**Authors:** Zhaoli Meng, Wei Fang, Mei Meng, Jicheng Zhang, Qizhi Wang, Guoqiang Qie, Man Chen, Chunting Wang

**Affiliations:** ^1^Department of Critical Care Medicine, Shandong Provincial Hospital, Cheeloo College of Medicine, Shandong University, Jinan, China; ^2^Department of Critical Care Medicine, Shandong Provincial Hospital Affiliated to Shandong First Medical University, Jinan, China; ^3^Department of Critical Care Medicine, Ruijin Hospital, Shanghai Jiao Tong University School of Medicine, Shanghai, China

**Keywords:** AFLP, maternal mortality, fetal mortality, risk factor, prognostic model

## Abstract

Acute fatty liver of pregnancy (AFLP) is a rare but potentially life-threatening hepatic disorder that leads to considerable maternal and fetal mortality. To explore the risk factors for maternal and fetal mortality in AFLP and develop new predictive models, through this retrospective study, we analyzed the demographic characteristics, clinical symptoms, and laboratory findings of 106 patients with AFLP who were admitted to Shandong Provincial Hospital. Risk factors for maternal and fetal mortality were analyzed by univariate and multivariate logistic regression analysis. The new models based on the multivariate logistic regression analysis and the model for end-stage liver disease (MELD) were tested in AFLP. The receiver operating characteristic curve (ROC) was applied to compare the predictive efficiency, sensitivity, and specificity of the two models. Prenatal nausea (*p* = 0.037), prolonged prothrombin time (*p* = 0.003), and elevated serum creatinine (*p* = 0.003) were independent risk factors for maternal mortality. The ROC curve showed that the area under the curve (AUC) of the MELD was 0.948, with a sensitivity of 100% and a specificity of 83.3%. The AUC of the new model for maternal mortality was 0.926, with a sensitivity of 90% and a specificity of 94.8%. Hepatic encephalopathy (*p* = 0.016) and thrombocytopenia (*p* = 0.001) were independent risk factors for fetal mortality. Using the ROC curve, the AUC of the MELD was 0.694, yielding a sensitivity of 68.8% and a specificity of 64.4%. The AUC of the new model for fetal mortality was 0.893, yielding a sensitivity of 100% and a specificity of 73.3%. Both the new predictive model for maternal mortality and the MELD showed good predictive efficacy for maternal mortality in patients with AFLP (AUC = 0.926 and 0.948, respectively), and the new predictive model for fetal mortality was superior to the MELD in predicting fetal mortality (AUC = 0.893 and 0.694, respectively). The two new predictive models were more readily available, less expensive, and easier to implement clinically, especially in low-income countries.

## Introduction

Acute fatty liver of pregnancy (AFLP) is a rare but potentially life-threatening hepatic disorder that occurs during the third trimester or early postpartum period. It is defined as severe hepatic synthetic dysfunction due to microvascular steatosis. Although the reported incidence of AFLP was 1 in 7,000 to 1 in 15,000 pregnancies (1), it could progress rapidly to serious complications such as disseminated intravascular coagulation (DIC), postpartum hemorrhage, multiple organ dysfunction syndrome (MODS), acute hepatic failure (AHF), and maternal or fetal death. The pathogenesis of AFLP remains unclear, and most of the literature supports that it is secondary to mitochondrial defects in the fetal long-chain 3-hydroxyacyl-coenzyme A dehydrogenase, as well as other enzymes potentially involved in fatty oxidation, leading to excessive accumulation of fatty acids in maternal hepatocytes, which, in turn, leads to lipotoxicity, oxidative damage, inflammation, and hepatocyte necrosis (2). Early recognition and diagnosis of AFLP with prompt termination of pregnancy and intensive supportive care are essential for both maternal and fetal survival. With advances in multidisciplinary supportive management of patients with AFLP, maternal and fetal mortality rates have decreased significantly to 7–18% and 9–23%, respectively (3).

Early assessment of the prognosis of patients with AFLP may play an important role in improving maternal and fetal survival (4), and predictive analytics for risk stratification can help to improve patients management in critical care setting and add some reference for this statement (5–7). Previous clinical studies on AFLP, largely based on a small number of patients owing to its low prevalence, have found significant differences in its epidemiology (1, 8), symptoms (9), complications (9), and outcomes (1, 10, 11). An excellent prediction model must be objective, easy to operate, and possess satisfactory sensitivity and specificity. The MELD, founded in 2000 by Malinchoc et al. of Mayo Clinic, the largest liver disease center in the United States, was a grading method for assessing the severity of end-stage liver disease. It was originally created to predict the survival of 231 patients with cirrhosis and portal hypertension after transjugular intrahepatic portosystemic shunt. The statistical model obtained by Cox proportional hazard regression identified four laboratory and clinical indicators that can be used to better assess the three-month survival of patients (12). Thereafter, Kamath et al. improved the scoring system to *R* = 3.8 ln (bilirubin) + 11.2 ln (INR) + 9.6 ln (creatinine) + 6.4 (13), which made the MELD one of the most widely used scoring systems for evaluating the prognosis of liver disease. Thus far, many studies have reported the ability of the MELD to predict the short-term prognosis of patients with acute liver failure and pregnancy-specific liver diseases (14, 15). However, large-sample studies have rarely investigated the efficacy of the MELD in predicting maternal and fetal outcome of AFLP, owing to the rarity of this condition. The existing literature predominantly consists of small hospital-based case series or historical cohorts identified retrospectively over a number of years. Therefore, there is an urgent need for clinical studies on AFLP, especially large-sample and multicenter prospective studies, to help clinicians make prognostic judgments.

Our study included 106 patients with AFLP who were admitted to our hospital during the past 10 years. We aimed to explore the independent risk factors for maternal and fetal mortality, and develop new models for predicting the poor prognosis of patients with AFLP.

## Patients and Methods

**Figure 1:**
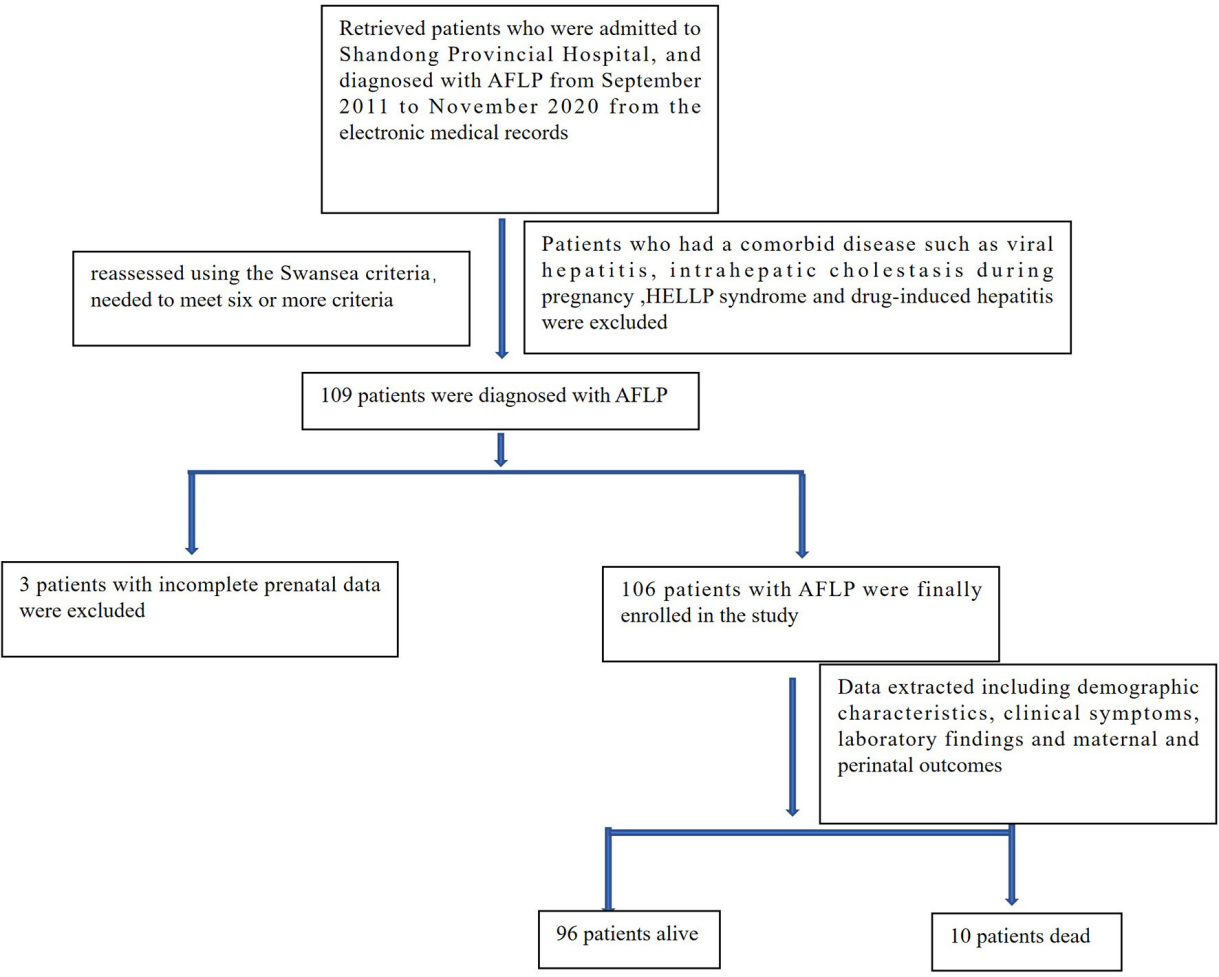
Flow chart of patient enrolment.

We retrospectively analyzed the data of 119 patients who were admitted to Shandong Provincial Hospital, Cheeloo College of Medicine, Shandong University and diagnosed with AFLP from September 2011 to November 2020. The diagnosis of all selected patients was reassessed using the Swansea criteria (wherein the diagnosis of AFLP requires the satisfaction of six or more criteria), as detailed in Table 1. Ten patients who also had a comorbid disease such as viral hepatitis, intrahepatic cholestasis during pregnancy; hemolysis, elevated liver enzymes, and low platelet (HELLP) syndrome; and drug-induced hepatitis, and three patients with incomplete prenatal data were excluded. A total of 106 patients with AFLP were finally enrolled in the study. The flowchart for patient enrollment is provided in Figure 1.

**Table 1:** Swansea criteria for diagnosis of AFLP.

**Variable**	**Finding**
Vomiting	Positive
Abdominal pain	Positive
Polydipsia or polyuria	Positive
Hepatic encephalopathy	Positive
Bilirubin	>14 μmol/L
Hypoglycemia	<4 mmol/L
Uric acid	>340 μmol/L
Leukocytosis	>11 × 10^9^/L
Ascites or ultrasound shows bright liver	Positive
ALT	>42 U/L
Serum ammonia	>47 μmol/L
Serum creatinine	>150 μmol/L
**Coagulopathy**	
PT	>14 s
APTT	34 s
Liver biopsy	Diffuse micro vesicular steatosis in hepatocytes

*ALT, alanine aminotransferase; PT, prothrombin time; APTT, activated partial thromboplastin time*.

*Vomiting: Nausea describes the unpleasant sensation of the imminent need to vomit, whereas vomiting refers to the forceful oral expulsion of gastric contents associated with contraction of the abdominal and chest wall musculature. Therefore, whereas nausea is a subjective experience, vomiting represents a physical event (16)*.

*Abdominal pain: pain of non-traumatic origin with a maximum duration of 5 days (17)*.

*Polydipsia: Primary polydipsia is a condition wherein there is excess consumption of fluids leading to polyuria with diluted urine and, ultimately, hyponatremia (18)*.

*Polyuria: production of more than 40–50 mL/kg of urine in a 24-h period (19, 20)*.

All patients were prenatally diagnosed with AFLP. Information regarding their laboratory findings, imaging data, and clinical symptoms was collected from the electronic medical records, and they were followed up within 1 month after discharge. The study was approved by the Biomedical Research Committee of Shandong Provincial Hospital (approval no. SWYX: NO.2021-052). The ethics committee waived the need for obtaining informed consent from the patients, because the study was an observational, retrospective study using a database from which the patients' identification information had been removed. Data extracted from these medical records included demographic characteristics, clinical symptoms, laboratory findings, clinical course, and maternal and perinatal outcomes.

Demographic characteristics included age, gestational weeks, parity, mode of delivery, single or twin fetus, fetal sex, admission to the intensive care unit (ICU) or not, and days from the first symptom to delivery. Clinical symptoms included abdominal pain, anorexia, nausea, vomiting, polyuria, jaundice, encephalopathy, and high blood pressure. Laboratory findings included prothrombin time (PT), activated partial thromboplastin time (APTT), international normalized ratio (INR), fibrinogen, white blood cell count, hemoglobin, percentage of neutrophils, neutrophils (N), platelet count (PLT), procalcitonin, blood urea nitrogen (BUN), blood creatinine (Cr), blood glucose, uric acid, aspartate aminotransferase (AST), alanine aminotransferase (ALT), gamma-glutamyl transpeptidase (GGT), alkaline phosphatase (ALP), total bilirubin (TBIL), direct bilirubin (DBIL), and albumin (Alb). Primary prognostic outcomes included maternal death and fetal death.

### Swansea Criteria for Diagnosis of AFLP

Owing to the rarity of AFLP, currently no internationally recognized diagnostic standard for this condition exists, except for liver biopsy. However, many studies have mentioned that although the gold standard for diagnosis is liver biopsy, this procedure is not always necessary, and the Swansea diagnostic criteria are regarded as an alternative to liver biopsy, as shown in Table 1 (11, 21, 22). Six or more criteria are required to diagnose AFLP. The exclusion criteria were as follows: viral hepatitis, intrahepatic cholestasis of pregnancy, HELLP syndrome, drug-induced hepatitis, autoimmune hepatitis, and other diseases.

### Statistical Analysis

GraphPad 8 and SPSS 21.0 software were used for statistical analysis. Continuous variables were expressed as mean and standard deviation, and categorical variables were expressed as count and percentages. Continuous variables were tested using Student's *t*-test, *t*-test with Welch correction, or Mann–Whitey *U*-test, depending on normal distribution and homogeneity in variance. The counting data were tested using the chi-square test. Univariate analysis was used to determine the factors related to maternal and fetal mortality. In order to avoid the omission of important factors, the significant variables obtained by univariate analysis and the variables with *p*-values between 0.05 and 0.1 or other clinically significant variables were included into the multiple logistic regression analysis performed by the forward selection method to determine the independent risk factors for different outcomes. Patients with severely incomplete data were excluded from the research. A small amount of missing data was not included in the univariate analysis; the results of univariate analysis showed that these variables with missing data were not related to maternal and fetal mortality. The data of the variables included in the multivariate analysis were complete. Independent risk factors, *B*-value, and constant obtained from the multiple logistic regression analysis were used to build prognostic prediction models. The new models and the MELD were used to assess all patients with AFLP. The receiver operating characteristic (ROC) curve was applied to compare the prediction efficiency, sensitivity, and specificity of the two models in evaluating the prognosis of patients with AFLP.

## Results

### Clinical Characteristics

A total of 106 patients with AFLP were enrolled in this study. Their demographic characteristics and clinical symptoms are shown in Table 2. Laboratory findings and prognostic outcomes are shown in Tables 3, 4, respectively. The average maternal age was 29.8 ± 4.8 years, and the average gestational age was 35.8 ± 2.9 weeks. The median duration from the first symptom to delivery was 7.9 ± 7.9 days. In total, 43 (40.6%) patients were primigravida and 63 (59.4%) were multigravida; 98 (92.5%) patients delivered by cesarean section and 8 (7.5%) patients delivered vaginally. A total of 91 (76.5%) male and 28 (23.5%) female infants were born. Among all patients with AFLP, 96 (90.6%) were admitted to the intensive care unit (ICU) after delivery. The common clinical symptoms included anorexia (56.6%), vomiting (48.1%), nausea (46.2%), abdominal pain (30.2%), jaundice (23.6%), hypertension (15.1%), polydipsia and polyuria (9.4%), and hepatic encephalopathy/disturbance of consciousness (7.5%).

**Table 2:** Demographic characteristics and clinical symptoms of patients with AFLP (*n* = 106).

**Variable**	**Mean ± SD/No. (%)**
**Demographic characteristics**	
Maternal age (year)	29.8 ± 4.8
**Gravidity**	
1	31 (29.2)
2	29 (27.4)
≥3	46 (43.4)
**Parity**	
1	43 (40.6)
2	54 (50.9)
3	9 (8.5)
**Delivery**	
Cesarean section	98 (92.5)
Vaginal	8 (7.5)
**Number of fetuses**	
Single	93 (87.7)
Twins	13 (12.3)
**Gender of baby**	
Female	24 (22.6)
Male	69 (65.1)
Female/male	2 (1.9)
Female/female	1 (0.9)
Male/male	10 (9.4)
Admitted to ICU	96 (90.6)
Days from the first symptom to delivery	7.9 ± 7.9
Gestational age (weeks) when the first symptom occurred	35.8 ± 2.9
**Symptoms**	
Abdominal pain	32 (30.2)
Anorexia	60 (56.6)
Nausea	49 (46.2)
Vomiting	51 (48.1)
Polydipsia/polyuria	10 (9.4)
Jaundice	25 (23.6)
Hepatic encephalopathy	8 (7.5)
Hypertension	16 (15.1)

*AFLP, acute fatty liver of pregnancy; ICU, intensive care unit; SD, standard deviation*.

**Table 3:** Laboratory findings of patients with AFLP (*n* = 106).

**Variable**	**Mean ± SD**	**Reference range**
Prothrombin time (s)	22.5 ± 15.9	10.7–14
Activated partial thromboplastin time (s)	53.8 ± 27.4	28–45
International normalized ratio (INR)	2.1 ± 2.1	0.8–1.2
Fibrinogen (g/L)	1.6 ± 1.3	1.75–4.35
Leukocyte (× 10^9^/L)	15.1 ± 5.6	3.5–9.5
Hemoglobin (g/L)	111.2 ± 24.4	130–175
Neutrophil% (%)	75.3 ± 8.5	40–75
Neutrophil (× 10^9^/L)	11.5 ± 4.8	1.8–6.3
Platelets (× 10^9^/L)	150.3 ± 7.4	125–350
Procalcitonin (ng/mL)	4.6 ± 11.3	0–0.05
Blood urea nitrogen (mmol/L)	8.0 ± 5.5	2.8–7.14
Creatinine (μmol/L)	169.9 ± 95.7	40–135
Glucose (mmol/L)	4.4 ± 2.0	3.9–6.3
Uric acid (μmol/L)	495.3 ± 157.5	208–428
Aspartate aminotransferase (U/L)	289.2 ± 270.8	15–40
Alanine aminotransferase (U/L)	292.2 ± 281.6	9–50
Glutamyl transpeptidase (U/L)	97.2 ± 63.9	10–60
Alkaline phosphatase (U/L)	414.0 ± 220.4	45–125
Total bilirubin (μmol/L)	134.1 ± 102.8	3.5–23.5
Direct bilirubin (μmol/L)	82.5 ± 60.9	0.5–6.5
Albumin (g/L)	28.2 ± 5.7	40–55

**Table 4:** Complications and outcomes of patients with AFLP (*n* = 106).

**Variable**	**No. (%)**
**Maternal complications**	
Acute kidney injury	71 (67.0)
Disseminated intravascular coagulation	30 (28.3)
Post-partum hemorrhage/wound seroma	29 (27.4)
Sepsis	28 (26.4)
Multiple organ dysfunction syndrome	30 (28.3)
Acute hepatic failure	24 (22.6)
**Maternal outcome**	
Death	10 (9.4)
**Fetal outcome**	
Death	16 (15.1)

Coagulation tests yielded obviously abnormal results, including prolonged PT (22.5 ± 15.9 s), APTT (53.8 ± 27.4 s), INR (2.1 ± 2.1), and decreased fibrinogen levels (1.6 ± 1.3 g/L). The results of blood routine tests showed increased leukocyte (15.1 ± 5.6 × 10^9^/L) and neutrophil (11.5 ± 4.8 × 10^9^/L) counts, decreased hemoglobin (111.2 ± 24.4 g/L), and normal PLT count (150.3 ± 7.4 × 10^9^/L). Procalcitonin was significantly increased (4.6 ± 11.3 ng/mL). Liver function tests revealed increased levels of ALT (292.2 ± 281.6 U/L), AST (289.2 ± 270.8 U/L), GGT (97.2 ± 63.9 U/L), ALP (414.0 ± 220.4 U/L), TBIL (134.1 ± 102.8 μmol/L), and DBIL (82.5 ± 60.9 μmol/L). Renal function was impaired, as evident from increased BUN (8.0 ± 5.5 mmol/L) and Cr (169.9 ± 95.7 μmol/L) levels. Abdominal ultrasound was performed for 71 patients, out of which 42 (59.2%) patients showed positive results. However, 35 patients did not undergo prenatal abdominal ultrasound. The maternal mortality rate was 9.4% (10/106) and the fetal mortality rate was 15.1% (16/106). Nine patients died from multiple organ dysfunction syndrome and one patient died from postpartum hemorrhage. The common severe complications included acute kidney injury (AKI; 67.0%), DIC (28.3%), MODS (28.3%), postpartum hemorrhage (27.4%), sepsis (26.4%), and AHF (22.6%).

### Risk Factors for Maternal Mortality, and the New Predictive Model

The distinction in demographic and clinical characteristics and laboratory findings between survivors and non-survivors is summarized in Table 5. Univariate analyses showed that maternal mortality was significantly related to nausea (*p* = 0.042), hepatic encephalopathy (*p* = 0.027), prolonged PT (*p* < 0.0001), prolonged APTT (*p* = 0.0009), increased INR (*p* < 0.0001), decreased fibrinogen (*p* = 0.004), increased leukocytes (*p* = 0.018), increased neutrophils (*p* = 0.012), thrombocytopenia (*p* = 0.0003), increased Cr (*p* = 0.002), increased TBIL (*p* = 0.006), increased DBIL (*p* = 0.024), and decreased Alb (*p* = 0.017).

**Table 5:** Comparison of demographic, clinical, and laboratory characteristics between maternal survivors and non-survivors.

**Variable**	**No. (%)**		
	**Alive (*n* = 96)**	**Dead (*n* = 10)**	***p-*value**
**Demographic characteristics**			
Maternal age (year)	29.7 ± 4.8	31.5 ± 4.7	0.245
Gravidity			0.307
1	29 (30.2)	2 (20.0)	
2	27 (28.1%)	2 (20.0)	
≥3	40 (41.7)	6 (60.0)	
Parity			0.239
1	40 (41.7)	3 (30.0)	
2	49 (51.0)	5 (50.0)	
3	7 (7.3)	2 (20.0)	
Delivery			0.560
Cesarean section	89 (92.7)	9 (90.0)	
Vaginal	7 (7.3)	1 (10.0)	
Number of fetuses			0.608
Single	83 (86.5)	10 (100.0)	
Twins	13 (13.5)	0	
Gender of baby			0.531
Female	20 (20.8)	4 (40.0)	
Male	63 (65.6)	6 (60.0)	
Female/male	2 (2.1)	0	
Female/female	1 (1.0)	0	
Male/male	10 (10.4)	0	
Delivery in other hospital			0.219
Yes	19 (19.8)	4 (40.0)	
No	77 (80.2)	6 (60.0)	
Admitted to ICU	86 (89.6)	10 (100.0)	0.593
**Symptoms**			
Abdominal pain	29 (30.2)	3 (30.0)	>0.9999
Anorexia	54 (56.3)	6 (60.0)	>0.9999
Nausea	41 (42.7)	8 (80.0)	0.042^*^
Vomiting	44 (45.8)	7 (70.0)	0.191
Polydipsia/polyuria	9 (9.4)	1 (10.0)	>0.9999
Jaundice	24 (25.0)	1 (10.0)	0.446
Encephalopathy	5 (5.2)	3 (30.0)	0.027^*^
Hypertension	14 (14.6)	2 (20.0)	0.645
Days from the first symptom to delivery	7.7 ± 7.9	9.1 ± 8.3	0.375
Days of pregnancy when the first symptom occurred	250.8 ± 20.9	247.4 ± 8.8	0.273
**Laboratory findings**			
Prothrombin time (s)	20.7 ± 14.8	39.4 ± 16.8	<0.0001^****^
Activated partial thromboplastin time (s)	50.8 ± 23.5	82.8 ± 43.3	0.0009^***^
International normalized ratio	1.9 ± 2.0	4.1 ± 2.1	<0.0001^****^
Fibrinogen (g/L)	1.6 ± 1.3	0.8 ± 0.5	0.004^**^
Leukocyte (× 10^9^/L)	14.6 ± 5.1	19.6 ± 7.6	0.018^*^
Hemoglobin (g/L)	111.1 ± 24.7	111.7 ± 22.0	0.776
Neutrophil %	75.1 ± 8.7	77.0 ± 5.5	0.500
Neutrophil (× 10^9^/L)	11.2 ± 4.5	15.3 ± 5.9	0.012^*^
Platelets (× 10^9^/L)	157.7 ± 72.6	79.2 ± 32.0	0.0003^***^
Procalcitonin (ng/mL)	4.8 ± 12.0	3.6 ± 3.5	0.985
Blood urea nitrogen (mmol/L)	7.7 ± 5.3	10.8 ± 6.1	0.077
Creatinine (mg/dL)	156.6 ± 76.1	297.1 ± 160.6	0.002^**^
Glucose (mmol/L)	4.3 ± 1.4	5.2 ± 5.0	0.182
Uric acid (μmol/L)	490.6 ± 151.6	540 ± 210.8	0.611
Aspartate aminotransferase (U/L)	295.5 ± 275.2	229.2 ± 227.5	0.308
Alanine aminotransferase (U/L)	301.5 ± 287.9	203.2 ± 201.3	0.237
Glutamyl transpeptidase (U/L)	97.51 ± 62.7	93.8 ± 78.1	0.545
Alkaline phosphatase (U/L)	416.6 ± 223.2	388.5 ± 199.6	0.979
Total bilirubin (μmol/L)	124.7 ± 92.7	224.1 ± 150.5	0.006^**^
Direct bilirubin (μmol/L)	78.1 ± 58.3	124.5 ± 72.3	0.024^*^
Albumin (g/L)	28.6 ± 5.7	24.2 ± 5.1	0.017^*^

* *p < 0.05*,

** *p < 0.01*,

*** *p < 0.001*,

**** *p < 0.0001*.

The above significant variables and BUN (*p* = 0.077) were included in the logistic regression analysis performed using the forward selection approach, in order to avoid missing important risk factors. The results of logistic regression analysis showed that nausea (*p* = 0.037), prolonged PT (*p* = 0.003), and increased Cr (*p* = 0.003) were independent risk factors for maternal mortality, as shown in Table 6. Based on these three variables, a new predictive model for maternal mortality was established using the following formula:


$$\begin{array}{c} 2.911\;\times \text{ Nausea}+0.07\;\times \text{ Prothrombin time}+0.011\;\times \text{ Creatinine}-8.86 \end{array}$$

**Table 6:** Analysis of independent risk factors for maternal death.

**Variable**	**B**	**S.E**.	**OR**	**95%CI**	***p*-value**
Nausea	2.911	1.398	18.376	1.186–284.707	0.037^*^
Prothrombin time	0.07	0.024	1.073	1.024–1.124	0.003^**^
Creatinine	0.011	0.004	1.012	1.004–1.019	0.003^**^
Constant	−8.86	2.218			

*SE, standard error; CI, confidence interval*.

* *p < 0.05*,

** *p < 0.01*.

**Figure 2:**
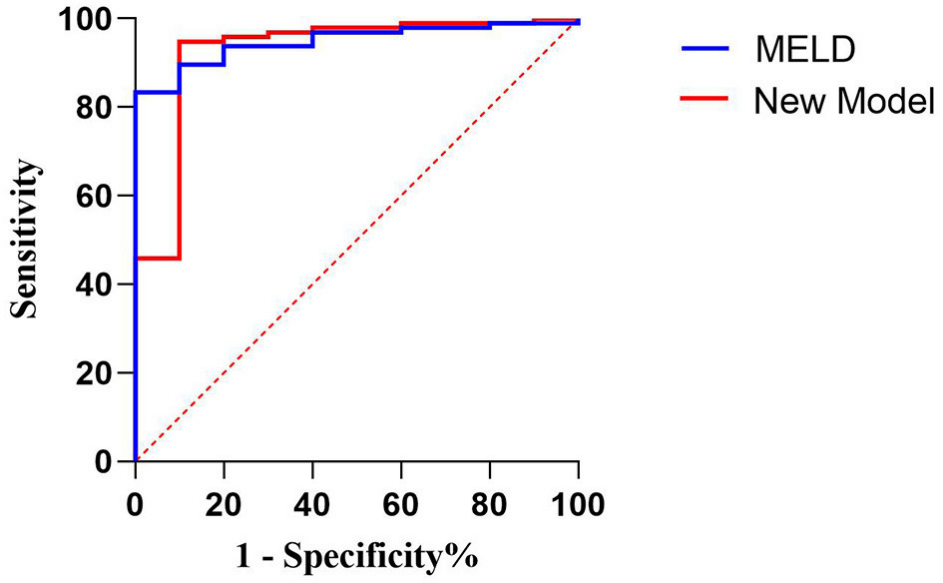
Receiver operating characteristic curve of the model for end-stage liver disease scoring system and the new model for predicting maternal death.

The ROC curve was used to evaluate the predictive efficiency of the new model and the MELD with regard to the prognosis of maternal mortality (Figure 2, Table 7). The threshold of the MELD was 29.835 and the AUC was 0.948, with a sensitivity of 100% and a specificity of 83.3%. The threshold of the new model was 0.186 and the AUC was 0.926, with a sensitivity of 90% and a specificity of 94.8%. Both the new model and the MELD could predict the prognosis of patients with AFLP. The new model was superior to the MELD in terms of specificity.

**Table 7:** Comparison of the two models for predicting maternal mortality.

**Model**	**Threshold**	**Sensitivity**	**Specificity**	**AUC**	**95% CI**
		**(%)**	**(%)**		
MELD	29.835	100	83.3	0.948	0.904–0.992
New model	0.186	90	94.8	0.926	0.825–1

*MELD, model for end-stage liver disease; AUC, area under the curve; CI, confidence interval*.

According to the threshold of the ROC curve, the MELD scores were divided into four groups and the new model scores were divided into three groups (Table 8). The mortality rate of the patients with AFLP gradually increased with the increase in the scores of the two models. When the MELD score was ≥40, the mortality rate was 57.14%; when the new model score was ≥0, the mortality rate was 66.67%. Therefore, the new model could more acutely predict maternal mortality. Moreover, the threshold, which was nearly 0, indicated that the new model was more convenient for clinical application.

**Table 8:** Relationship between MELD score and the new model for maternal mortality.

**MELD score**	***n***	**Dead**	**Maternal mortality (%)**	**New model score**	***n***	**Dead**	**Maternal mortality (%)**
≥40	7	4	57.14	≥0	6	4	66.67
≥30, <40	18	5	27.78	≥−3, <0	28	5	17.86
≥20, <30	46	1	2.17	< −3	72	1	1.39
<20	35	0	0.00				

*MELD, model for end-stage liver disease*.

### Risk Factors for Fetal Mortality, and the New Predictive Model

As shown in Table 9, univariate analysis showed that fetal mortality was significantly related to encephalopathy (*p* = 0.017), prolonged PT (*p* = 0.0005), prolonged APTT (*p* < 0.0001), increased INR (*p* = 0.0008), decreased fibrinogen (*p* = 0.016), elevated leukocyte (*p* = 0.043), thrombocytopenia (*p* < 0.0001), decreased GGT (*p* = 0.019), increased TBIL (*p* = 0.007), increased DBIL (*p* = 0.018), and decreased Alb (*p* = 0.006).

**Table 9:** Comparison of demographic, clinical, and laboratory characteristics between fetal survivors and non-survivors.

**Variable**	**No. (%)**		
	**Alive (*n* = 90)**	**Dead (*n* = 16)**	***p-*value**
**Demographic characteristics**			
Maternal age (year)	29.6 ± 4.7	31.1 ± 5.6	0.275
Gravidity			0.258
1	28 (31.1)	3 (18.7)	
2	22 (24.4)	7 (43.8)	
≥3	40 (44.5)	6 (37.5)	
Parity			0.299
1	39 (43.3)	4 (25.0)	
2	43 (47.8)	11 (68.8)	
3	8 (8.9)	1 (6.2)	
Delivery			0.099
Cesarean section	85 (94.4)	13 (81.3)	
Vaginal	5 (5.6)	3 (18.7)	
Number of fetuses			0.411
Single	80 (88.9)	13 (81.3)	
Twins	10 (11.1)	3 (18.7)	
Gender of baby			0.531
Female	22 (24.4)	11 (68.8)	
Male	58 (64.4)	2 (12.5)	
Female/male	2 (2.2)	0	
Female/female	7 (7.8)	3 (18.7)	
Male/male	1 (1.1)	0	
Admitted to ICU	81 (90.0)	15 (93.8)	>0.999
**Symptoms**			
Abdominal pain	28 (31.1)	4 (25.0)	0.772
Anorexia	50 (55.6)	10 (62.5)	0.785
Nausea	41 (45.6)	8 (50.0)	0.790
Vomiting	43 (47.8)	8 (50.0)	>0.999
Polydipsia/polyuria	10 (11.1)	0	0.353
Jaundice	21 (23.3)	4 (25.0)	>0.999
Encephalopathy	4 (4.4)	4 (25.0)	0.017^*^
Hypertension	14 (15.2)	2 (12.5)	>0.999
Days from the first symptom to delivery	7.4 ± 7.2	10.8 ± 10.7	0.394
Days of pregnancy when the first symptom occurred	251.0 ± 19.5	242.6 ± 19.2	0.051
**Laboratory findings**			
Prothrombin time (s)	20.8 ± 13.0	31.9 ± 25.3	0.0005^***^
Activated partial thromboplastin time (s)	48.9 ± 19.7	81.4 ± 44.6	<0.0001^****^
International normalized ratio	1.9 ± 1.5	3.3 ± 4.1	0.0008^***^
Fibrinogen (g/L)	1.7 ± 1.3	1.1 ± 1.0	0.016^*^
Leukocyte (× 10^9^/L)	14.8 ± 5.7	17.0 ± 4.2	0.043^*^
Hemoglobin (g/L)	112.6 ± 24.1	102.9 ± 25.0	0.141
Neutrophil %	75.0 ± 8.6	76.9 ± 7.5	0.419
Neutrophil (× 10^9^/L)	11.3 ± 4.9	13.0 ± 3.9	0.087
Platelets (× 10^9^/L)	164.2 ± 70.3	72.2 ± 28.1	<0.0001^****^
Procalcitonin (ng/mL)	3.2 ± 2.9	10.8 ± 24.9	0.074
Blood urea nitrogen (mmol/L)	7.7 ± 4.5	10.0 ± 9.1	0.422
Creatinine (mg/dL)	163.3 ± 83.9	206.4 ± 143.7	0.259
Glucose (mmol/L)	4.4 ± 2.0	4.3 ± 1.8	0.778
Uric acid (μmol/L)	496.4 ± 141.6	488.7 ± 233.5	0.463
Aspartate aminotransferase (U/L)	254.9 ± 183.2	309.5 ± 232.1	0.468
Alanine aminotransferase (U/L)	299.1 ± 289.8	253.7 ± 234.5	0.485
Glutamyl transpeptidase (U/L)	101.4 ± 63.0	73.1 ± 65.6	0.019^*^
Alkaline phosphatase (U/L)	428.3 ± 226.6	333.1 ± 164.8	0.131
Total bilirubin (μmol/L)	122.2 ± 91.5	201.3 ± 136.4	0.007^**^
Direct bilirubin (μmol/L)	76.1 ± 56.2	118.1 ± 75.0	0.018^*^
Albumin (g/L)	28.9 ± 5.5	24.6 ± 5.5	0.006^**^

* *p < 0.05*,

** *p < 0.01*,

*** *p < 0.001*,

**** *p < 0.0001*.

Multivariate logistic regression analysis showed that encephalopathy (*p* = 0.016) and thrombocytopenia (*p* = 0.001) were independent risk factors for fetal mortality (Table 10). Thereafter, a new predictive model for fetal mortality was established using the following formula:


$$\begin{array}{c} 2.411\;\times \text{ encephalopathy}-0.44\;\times \text{ platelets}+2.506 \end{array}$$

**Table 10:** Analysis of independent risk factors for fetal mortality.

**Variable**	**B**	**S.E**.	**OR**	**95%CI**	***p*-value**
Encephalopathy	2.411	0.999	11.141	1.574–78.87	0.016^*^
Platelets	−0.44	0.013	0.957	0.933–0.981	0.001^**^
Constant	2.506	1.087			

*SE, standard error; CI, confidence interval*.

* *p < 0.05*,

** *p < 0.01*.

**Figure 3:**
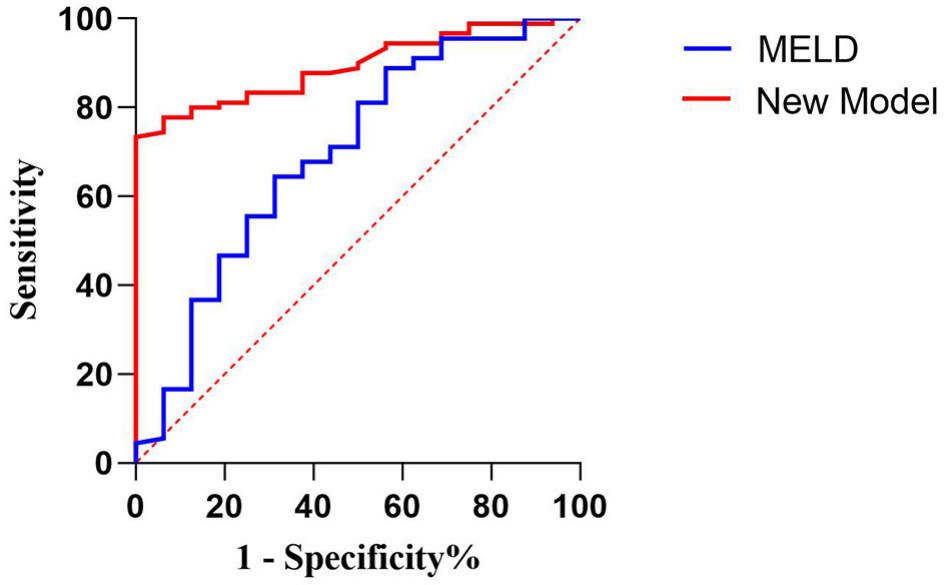
Receiver operating characteristic curve of the model for end-stage liver disease scoring system and the new model for predicting fetal death.

In predicting fetal mortality, the threshold of the MELD was 25.124 and the AUC was 0.694, with a sensitivity of 68.8% and a specificity of 64.4%. The threshold of the new model was −45.234 and the AUC was 0.893, with a sensitivity of 100% and a specificity of 73.3%. Thus, compared with the MELD, the new model could more accurately predict fetal mortality, with a higher sensitivity and specificity (Table 11, Figure 3).

**Table 11:** Comparison of the two models for predicting fetal mortality.

**Model**	**Threshold**	**Sensitivity**	**Specificity**	**AUC**	**95% CI**
		**(%)**	**(%)**		
MELD	25.124	68.8	64.4	0.694	0.543–0.846
New model	−45.234	100	73.3	0.893	0.832–0.955

*MELD, model for end-stage liver disease; AUC, area under the curve; CI, confidence interval*.

Both models were grouped according to the previous method (Table 12). The fetal mortality of patients with AFLP also gradually increased with the increase in the scores of the two models. When the MELD score was ≥40, the fetal mortality rate was 50%; when the new model score was ≥−50 and < −5„ the fetal mortality rate was 38.1%. Although the fetal mortality rate was lower when the new model score exceeded the threshold, it could predict the adverse outcomes of all fetuses, which indicated the high sensitivity and clinical application value of this model.

**Table 12:** Relationship between MELD score and the new model for fetal mortality.

**MELD score**	***n***	**Dead**	**Fetal mortality (%)**	**New model score**	***n***	**Dead**	**Fetal mortality (%)**
≥40	8	4	50.00	≥−50, < −5	42	16	38.10
≥30, <40	18	4	22.22	≥−100, < −50	43	0	0.00
≥20, <30	45	6	13.33	< −100	21	0	0.00
<20	35	2	5.71				

*MELD, model for end-stage liver disease*.

## Discussion

AFLP is a rare and fatal obstetric emergency that occurs in the second and third trimester of pregnancy or in the early postpartum period. It can lead to acute liver failure, AKI, multiple organ failure, and even maternal and fetal death. Many studies have analyzed the high-risk factors for the morbidity associated with AFLP, fatal complications, and perinatal death. Recent studies have shown that being a primigravida, multiple pregnancies, carrying a male fetus, other liver diseases during pregnancy, previous history of AFLP, and pre-eclampsia are the potential risk factors for AFLP (1, 23–25). The recognition of high-risk factors is helpful for the prevention and treatment of AFLP, and can consequently improve the prognosis of the mother and the child. Early diagnosis; prompt delivery; and multidisciplinary supportive care from the departments of obstetrics, blood transfusion, and the ICU have resulted in improved maternal mortality (3). Although liver biopsy is the gold standard for the diagnosis of AFLP, it is rarely performed owing to its invasive nature and because it can cause complications in the presence of coagulopathy. In addition, liver biopsy is just a diagnostic method and does not contribute significantly to the treatment of AFLP. Therefore, none of the patients with AFLP in this study underwent liver biopsy.

The 106 patients with AFLP who were enrolled in this study delivered 119 fetuses, including 13 twin pregnancies and 93 single pregnancies. The incidence of twin pregnancy was 12.3% (13/106), which occurred only in the surviving group; however, there was no statistically significant difference in the incidence of twin pregnancy between the survivor and non-survivor groups (13.5% vs. 0, *p* = 0.608). This finding is similar to the results of another retrospective study conducted in China by Cheng et al. (26) that showed that the incidence of twin pregnancy among patients with AFLP was 28.1%; however, there was a statistically significant difference between the survivor and non-survivor groups (44.4 vs. 7.1%, *p* = 0.02). This indicated that twin pregnancy may be a potential protective factor for patients with AFLP; however, this is contrary to the results of the prospective study conducted by Knight et al. (1). Although our study enrolled the largest number of patients among the three studies, it was still not a sufficiently large sample. Because of the rarity of AFLP, our study does not have the power to determine whether this is a statistically significant relationship or just a chance finding. A previous study by Gao et al. showed that male fetus, intrauterine death, postpartum diagnosis of AFLP, DIC, and prolonged PT and APTT were potential risk factors for maternal mortality in AFLP, whereas a history of legal termination of pregnancy, and increased TBIL and serum Cr were independent risk factors (27). In this study, male fetuses (*p* = 0.580) and a history of legal termination of pregnancy (*p* = 0.239) showed no statistically significant difference between the two groups and were not included in the potential risk factors for maternal mortality in AFLP.

Previous studies have rarely included a predictive model for fetal mortality. In this study, a new model for predicting fetal mortality was established and the predictive value of the MELD was also verified. The results of multivariate logistic regression analysis indicated that hepatic encephalopathy (*p* = 0.016) and thrombocytopenia (*p* = 0.001) were independent risk factors for fetal mortality in patients with AFLP. Hepatic encephalopathy is a comprehensive disorder of central nervous system dysfunction caused by severe liver disease. As the most direct complication of liver damage in patients with acute liver failure, it is one of the causes of death in patients with liver disease. Its occurrence suggests that patients with AFLP have had acute liver failure before delivery and the fetus has a high incidence of intrauterine distress and stillbirth. In patients with pre-eclampsia and HELLP syndrome, thrombocytopenia is an independent risk factor for postpartum complications such as infection, thromboembolism, and DIC, and these complications are also common in patients with AFLP (28). The retrospective study by Cheng et al. showed that carrying a male fetus and vaginal delivery were risk factors for fetal mortality; however, these two variables did not show significant positive predictive value in our study (26). Gao et al. found that fetal distress and prolonged APTT were risk factors for fetal mortality (27). The univariate analysis in our study showed that prolonged APTT was a risk factor for fetal mortality (*p* < 0.0001), but multivariate analysis showed no positive predictive value. The new predictive model for fetal mortality based on hepatic encephalopathy and thrombocytopenia was compared with the MELD with regard to the prediction of fetal mortality. The results showed that, compared with the MELD, the new predictive model for fetal mortality could more accurately predict fetal mortality (AUC = 0.893 and 0.694, respectively), with a higher sensitivity and specificity, and the variables were fewer, more readily available, and less expensive.

In the present study, the common severe complications besides death were AKI (67.0%), DIC (28.3%), MODS (28.3%), postpartum hemorrhage (27.4%), sepsis (26.4%), and AHF (22.6%), which is consistent with the results of the study by Chen et al. (29). In their study, the most common maternal complication was acute renal dysfunction (79.5%), followed by DIC (47.7%) and MODS (38.6%).

Maternal and fetal mortality rates attributable to AFLP vary greatly among studies, with the maternal mortality rate ranging from 12 to 18% and the fetal mortality rate ranging from 7 to 58% (30). Our previous clinical study showed that the maternal and fetal mortality rates of 52 patients with AFLP admitted to our hospital from January 2001 to December 2011 were 8% and 23%, respectively (31). In this study, a total of 106 patients with AFLP were admitted to our hospital from September 2011 to November 2020, and 119 fetuses were delivered. The maternal and fetal mortality rates were 9.4% (10/106) and 15.1% (18/119) respectively, both of which were lower than those reported in other studies. Timely termination of pregnancy is crucial in the treatment and a basic principle, because AFLP is an idiopathic disease during pregnancy. In the last decade, in our hospital, 83 (78.3%) pregnancies were terminated within 24 h when AFLP was strongly suspected or diagnosed, and 96 (90.6%) patients were admitted to the ICU after delivery. We attributed the lower maternal and fetal mortality to the proactive policy of early diagnosis and prompt termination, and close cooperation between obstetricians and critical care physicians. Compared with the last decade, the maternal mortality rate has declined slightly and the fetal mortality rate has decreased significantly in our hospital. This may be related to the loosening of the two-child policy, leading to an increasing number of older mothers and, consequently, more complications during pregnancy, causing a slight increase in maternal mortality. However, with the development of multidisciplinary supportive management in our hospital, especially pediatric intensive care, the level of comprehensive treatment of the fetus has been greatly improved, leading to a significant decline in the fetal mortality rate.

In this study, prenatal nausea (*p* = 0.037), prolonged PT (*p* = 0.003), and elevated serum Cr (p = 0.003) were independent risk factors for maternal mortality in patients with AFLP. Another study reported that ascites, thrombocytopenia, and serum Cr were independent risk factors for postpartum complications in pre-eclampsia and HELLP syndrome (32). The clinical symptoms of AFLP are similar to those of HELLP syndrome, and both belong to pregnancy-specific liver diseases. The predictive model for AFLP also included one clinical symptom and two laboratory findings, and elevated serum Cr was an independent risk factor for both AFLP and HELLP syndrome. However, the difference between the PLT count in AFLP was statistically significant in univariate analysis (*p* = 0.0003) and was eliminated in multivariate logistic regression analysis. This suggests that thrombocytopenia is a potential risk factor for maternal mortality in AFLP, which needs to be verified by a larger-sample study. Transaminase levels have not been shown to be important across most disease models in liver disease, including our model for AFLP (*p* > 0.05). A single-center retrospective study with 130 cases (AFLP = 32; HELLP = 81; pre-eclampsia and liver disease = 17) showed that both the MELD and the new model with two objective variables, namely serum TBIL and INR, were reliable for predicting the short-term mortality in patients with pregnancy-specific liver disease (followed up until 3 months after delivery or until death) (15). In the present study, TBIL and INR were statistically significant in univariate analysis (*p* = 0.006 and *p* < 0.0001, respectively), but they were eliminated in multivariate logistic regression analysis, which also suggested that increased TBIL and prolonged INR are potential risk factors for maternal mortality in AFLP; further prospective studies with larger sample sizes are warranted to explore the risk factors for maternal mortality in patients with AFLP.

Previous clinical studies have shown that the MELD, based on TBIL, Cr, and INR shows good predictive efficacy for acute liver failure and pregnancy-specific liver disease (14, 15). A study conducted in China showed that the MELD was a good predictor of all complications of AFLP, including ascites, hepatic encephalopathy, sepsis, and renal insufficiency (all AUCs > 0.8), and the optimal cut-off values were close to 30 (33). Our study also verified that both the MELD and the new model show good predictive efficacy in predicting maternal mortality in AFLP (AUC = 0.948 and 0.926, respectively).

Overall, compared with previous models based on laboratory findings alone, the new predictive model for maternal mortality included one clinical symptom and two laboratory findings, making it more readily available, less expensive, and easier to implement clinically. To the best of our knowledge, the symptom of nausea that we identified as an independent risk factor for AFLP has not been previously described.

This study had a long duration of almost 10 years, and is the largest single-center clinical study on AFLP so far. The number of patients with AFLP enrolled in this study is only second to that in the multicenter study by Gao et al. in which our hospital has participated in the past (27). As all patients with AFLP came from one single center, they received similar obstetric and multidisciplinary treatments after hospitalization, and some limitations of different medical levels were counter-balanced.

There are some limitations to our research. Firstly, we did not evaluate the morbidity of AFLP owing to the deficiency of data on total pregnant women during the study period. Secondly, this was a single-center and small-sample study because of the rarity of AFLP, which might reduce the general applicability of our findings, although we had extended the study period to almost one decade and our study was a retrospective study. Thirdly, as Shandong Provincial Hospital is a tertiary referral center for critical patients in China, some patients with AFLP were referred to our hospital after severe postpartum complications, and their condition was relatively critical. The manner and timing of medical intervention during their prenatal treatment differed, which directly affected the prognosis of the patients. Finally, it is noteworthy that the AUROC value is inflated in the internal data because external validation was not performed in the study.

In summary, both the new predictive model for maternal mortality and the MELD showed good predictive efficacy for maternal mortality in patients with AFLP (AUC = 0.926 and 0.948, respectively), and the new predictive model was superior to the MELD in predicting fetal mortality (AUC = 0.893 and 0.694, respectively). The two new predictive models were more readily available, less expensive, and easier to implement clinically, especially in low-income countries. However, as our study is a single-center, retrospective study with a small sample size, further prospective studies with large sample sizes are warranted to evaluate the efficiency of these two models for predicting maternal and fetal mortality in patients with AFLP.

## Data Availability Statement

The raw data supporting the conclusions of this article will be made available by the authors, without undue reservation.

## Ethics Statement

The studies involving human participants were reviewed and approved by the Biomedical Research Committee of Shandong Provincial Hospital (approval no. SWYX: NO.2021-052). Written informed consent for participation was not required for this study in accordance with the national legislation and the institutional requirements.

## Author Contributions

CW, MC, and ZM contributed to the conception and design of the study. CW and WF organized the database. MC performed the statistical analysis. ZM wrote the first draft of the manuscript. MM, JZ, QW, and GQ revised the sections of the manuscript. All authors contributed to manuscript revision, read, and approved the submitted version.

## Conflict of Interest

The authors declare that the research was conducted in the absence of any commercial or financial relationships that could be construed as a potential conflict of interest.

## Publisher's Note

All claims expressed in this article are solely those of the authors and do not necessarily represent those of their affiliated organizations, or those of the publisher, the editors and the reviewers. Any product that may be evaluated in this article, or claim that may be made by its manufacturer, is not guaranteed or endorsed by the publisher.
