# Corrigendum: Risk Factors for Maternal and Fetal Mortality in Acute Fatty Liver of Pregnancy and New Predictive Models

**DOI:** 10.3389/fmed.2021.786395

**Published:** 2021-10-19

**Authors:** Zhaoli Meng, Wei Fang, Mei Meng, Jicheng Zhang, Qizhi Wang, Guoqiang Qie, Man Chen, Chunting Wang

**Affiliations:** ^1^Department of Critical Care Medicine, Shandong Provincial Hospital, Cheeloo College of Medicine, Shandong University, Jinan, China; ^2^Department of Critical Care Medicine, Shandong Provincial Hospital Affiliated to Shandong First Medical University, Jinan, China; ^3^Department of Critical Care Medicine, Ruijin Hospital, Shanghai Jiao Tong University School of Medicine, Shanghai, China

**Keywords:** AFLP, maternal mortality, fetal mortality, risk factor, prognostic model

In the published article, there was an error in affiliation 1. Instead of “Department of Critical Care Medicine, Cheeloo College of Medicine, Shandong Provincial Hospital, Shandong University, Jinan, China,” it should be “Department of Critical Care Medicine, Shandong Provincial Hospital, Cheeloo College of Medicine, Shandong University, Jinan, China.”

The authors apologize for this error and state that this does not change the scientific conclusions of the article in any way. The original article has been updated.

## Publisher's Note

All claims expressed in this article are solely those of the authors and do not necessarily represent those of their affiliated organizations, or those of the publisher, the editors and the reviewers. Any product that may be evaluated in this article, or claim that may be made by its manufacturer, is not guaranteed or endorsed by the publisher.

